# Supplementary material for: Analysis of the efficacy of splenic artery superselective embolization in cirrhosis with hepatocellular carcinoma
Source: PLoS One. 2025 May 28;20(5):e0323829. doi: 10.1371/journal.pone.0323829 (PMC12119008; doi:10.1371/journal.pone.0323829)
Supplement: S1 File — (DOCX) [file pone.0323829.s001.docx]

**Supplementary material for Manuscript No.:** PONE-D-24-23322

**Title: Analysis of the efficacy of splenic artery superselective embolization in cirrhosis with hepatocellular carcinoma**

**Table S1.** The raw data of patients.

| Patient id | time | WBC | RBC | PLT | group |
| --- | --- | --- | --- | --- | --- |
| 1 | 1week | 2 | 4.16 | 49 | 1 |
|  | 2w | 4.57 | 4.04 | 54 |  |
|  | 5w | 4.36 | 3.78 | 65 |  |
|  | 13w | 4.23 | 3.68 | 59 |  |
|  | 25w | 4.56 | 3.54 | 53 |  |
|  | | | | | |
| 2 | 1w | 3.4 | 2.68 | 51 | 1 |
|  | 2w | 11.34 | 2.78 | 57 |  |
|  | 5w | 3.78 | 2.89 | 58 |  |
|  | 13w | 3.31 | 2.85 | 53 |  |
|  | 25w | 4.38 | 2.34 | 50 |  |
|  | | | | | |
| 3 | 1w | 2.33 | 3.39 | 40 | 1 |
|  | 2w | 3.88 | 3.66 | 58 |  |
|  | 5w | 4.14 | 3.92 | 122 |  |
|  | 13w | 7.34 | k3.23 | 185 |  |
|  | 25w | 9.24 | 2.88 | 138 |  |
|  |  |  |  |  |  |
| 4 | 1w | 1.38 | 4.25 | 67 | 1 |
|  | 2w | 1.77 | 3.86 | 67 |  |
|  | 5w | 4.26 | 4.05 | 118 |  |
|  | 13w | 3.78 | 3.96 | 105 |  |
|  | 25w | 4.77 | 3.95 | 110 |  |
|  | | | | | |
| 5 | 1w | 3.88 | 4.26 | 61 | 1 |
|  | 2w | 9.59 | 4 | 152 |  |
|  | 5w | 3.62 | 4.23 | 116 |  |
|  | 13w | 6.87 | 3.88 | 138 |  |
|  | 25w | 6.56 | 3.76 | 127 |  |
|  | | | | | |
| 6 | 1w | 4.55 | 3.7 | 61 | 1 |
|  | 2w | 9.09 | 3.65 | 84 |  |
|  | 5w | 8.17 | 3.55 | 115 |  |
|  | 13w | 8.6 | 3.95 | 152 |  |
|  | 25w | 10.3 | 3.73 | 146 |  |
|  | | | | | |
| 7 | 1w | 3.19 | 3.02 | 14 | 1 |
|  | 2w | 5.36 | 3.29 | 81 |  |
|  | 5w | 4.81 | 3.33 | 124 |  |
|  | 13w | 4.81 | 3.13 | 165 |  |
|  | 25w | 5.27 | 3.24 | 150 |  |
|  | | | | | |
| 8 | 1w | 1.24 | 3.41 | 44 | 1 |
|  | 2w | 5.78 | 3.3 | 71 |  |
|  | 5w | 8.8 | 3.08 | 48 |  |
|  | 13w | 2.29 | 3.67 | 56 |  |
|  | 25w | 3.32 | 3.59 | 73 |  |
|  | | | | | |
| 9 | 1w | 2.77 | 2.91 | 17 | 1 |
|  | 2w | 5.69 | 2.61 | 74 |  |
|  | 5w | 5.73 | 2.34 | 65 |  |
|  | 13w | 4.55 | 2.47 | 67 |  |
|  | 25w | 4.76 | 2.36 | 60 |  |
|  | | | | | |
| 10 | 1w | 4.07 | 5.03 | 24 | 1 |
|  | 2w | 9.17 | 4.84 | 107 |  |
|  | 5w | 5.11 | 5.41 | 70 |  |
|  | 13w | 6.73 | 5.56 | 87 |  |
|  | 25w | 5.18 | 5.87 | 99 |  |
|  | | | | | |
| 11 | 1w | 1.37 | 4.22 | 26 | 1 |
|  | 2w | 2.17 | 3.84 | 39 |  |
|  | 5w | 2.43 | 3.57 | 35 |  |
|  | 13w | 2.23 | 3.95 | 37 |  |
|  | 25w | 2.87 | 3.64 | 33 |  |
|  |  |  |  |  |  |
| 12 | 1w | 2.28 | 4.07 | 38 | 1 |
|  | 2w | 6.86 | 4.61 | 67 |  |
|  | 5w | 3.16 | 4.73 | 95 |  |
|  | 13w | 2.18 | 3.89 | 93 |  |
|  | 25w | 2.81 | 3.92 | 51 |  |
|  | | | | | |
| 13 | 1w | 4.18 | 4.44 | 48 | 1 |
|  | 2w | 9.93 | 4.69 | 64 |  |
|  | 5w | 6.89 | 4.32 | 80 |  |
|  | 13w | 4.57 | 3.67 | 105 |  |
|  | 25w | 6.21 | 2.93 | 122 |  |
|  |  |  |  |  |  |
| 14 | 1w | 2.69 | 3.08 | 67 | 1 |
|  | 2w | 3.94 | 3.42 | 51 |  |
|  | 5w | 2.02 | 3 | 55 |  |
|  | 13w | 1.9 | 2.41 | 62 |  |
|  | 25w | 3.39 | 2.92 | 75 |  |
|  | | | | | |
| 15 | 1w | 1.55 | 3.07 | 59 | 1 |
|  | 2w | 6.5 | 3.03 | 57 |  |
|  | 5w | 4.15 | 2.98 | 121 |  |
|  | 13w | 3.98 | 2.92 | 137 |  |
|  | 25w | 9.21 | 2.48 | 138 |  |
|  | | | | | |
| 16 | 1w | 1.54 | 3.78 | 39 | 1 |
|  | 2w | 2.38 | 4.03 | 42 |  |
|  | 5w | 2.14 | 4.66 | 49 |  |
|  | 13w | 5.3 | 4.02 | 50 |  |
|  | 25w | 3.13 | 4.22 | 51 |  |
|  | | | | | |
| 17 | 1w | 4.62 | 3.35 | 95 | 1 |
|  | 2w | 5.1 | 3.18 | 163 |  |
|  | 5w | 2.43 | 3.12 | 187 |  |
|  | 13w | 3.92 | 4.1 | 170 |  |
|  | 25w | 7.37 | 4.24 | 251 |  |
|  | | | | | |
| 18 | 1w | 3.75 | 2.4 | 34 | 1 |
|  | 2w | 4.81 | 2.74 | 57 |  |
|  | 5w | 2.95 | 2.77 | 53 |  |
|  | 13w | 4.13 | 2.88 | 58 |  |
|  | 25w | 3.41 | 2.42 | 40 |  |
|  | | | | | |
| 19 | 1w | 0.9 | 2.58 | 58 | 1 |
|  | 2w | 4.7 | 2.89 | 128 |  |
|  | 5w | 5.09 | 2.73 | 138 |  |
|  | 13w | 3.51 | 2.68 | 107 |  |
|  | 25w | 3.76 | 2.58 | 87 |  |
|  | | | | | |
| 20 | 1w | 1.57 | 4.14 | 24 | 1 |
|  | 2w | 9.56 | 4.31 | 56 |  |
|  | 5w | 8.11 | 4.36 | 138 |  |
|  | 13w | 8.45 | 4.23 | 111 |  |
|  | 25w | 7.53 | 4.36 | 102 |  |
|  | | | | | |
| 21 | 1w | 2.32 | 3.46 | 34 | 1 |
|  | 2w | 4.83 | 3.49 | 46 |  |
|  | 5w | 3.54 | 2.85 | 61 |  |
|  | 13w | 6.67 | 2.93 | 60 |  |
|  | 25w | 6.13 | 2.72 | 30 |  |
|  | | | | | |
| 22 | 1w | 3.48 | 3.31 | 57 | 1 |
|  | 2w | 6.05 | 3.61 | 83 |  |
|  | 5w | 4.79 | 3.74 | 178 |  |
|  | 13w | 4.05 | 3.37 | 185 |  |
|  | 25w | 5.68 | 3.56 | 123 |  |
|  | | | | | |
| 23 | 1w | 2.52 | 2.7 | 74 | 1 |
|  | 2w | 5.17 | 3.13 | 141 |  |
|  | 5w | 2.23 | 2.56 | 72 |  |
|  | 13w | 5.83 | 2.94 | 133 |  |
|  | 25w | 4.88 | 3.36 | 82 |  |
|  | | | | | |
| 24 | 1w | 5.14 | 3.1 | 71 | 1 |
|  | 2w | 6.74 | 2.75 | 144 |  |
|  | 5w | 8.91 | 2 | 88 |  |
|  | 13w | 8.93 | 2.51 | 114 |  |
|  | 25w | 7.69 | 3.34 | 93 |  |
|  | | | | | |
| 25 | 1w | 2.49 | 3.42 | 42 | 1 |
|  | 2w | 3.81 | 3.38 | 104 |  |
|  | 5w | 3.09 | 3.1 | 114 |  |
|  | 13w | 3.64 | 3.2 | 68 |  |
|  | 25w | 5.67 | 3.43 | 63 |  |
|  | | | | | |
| 26 | 1w | 3.14 | 3.82 | 64 | 1 |
|  | 2w | 8.19 | 4.49 | 107 |  |
|  | 5w | 8.9 | 3.36 | 147 |  |
|  | 13w | 5.66 | 3.2 | 139 |  |
|  | 25w | 10.31 | 2.8 | 101 |  |
|  | | | | | |
| 27 | 1w | 3.34 | 4.31 | 32 | 1 |
|  | 2w | 5.59 | 3.85 | 36 |  |
|  | 5w | 4.8 | 4.47 | 39 |  |
|  | 13w | 4.62 | 4.06 | 45 |  |
|  | 25w | 4.46 | 4.09 | 39 |  |
|  | | | | | |
| 28 | 1w | 1.83 | 3.49 | 35 | 1 |
|  | 2w | 3.84 | 3.43 | 82 |  |
|  | 5w | 2.81 | 4.09 | 80 |  |
|  | 13w | 5.02 | 4.06 | 78 |  |
|  | 25w | 7.95 | 3.07 | 92 |  |
|  | | | | | |
| 29 | 1w | 2.05 | 3.3 | 30 | 1 |
|  | 2w | 3.29 | 3.07 | 60 |  |
|  | 5w | 2.98 | 4.78 | 38 |  |
|  | 13w | 3.39 | 4.15 | 57 |  |
|  | 25w | 2.53 | 4.44 | 47 |  |
|  | | | | | |
| 30 | 1w | 2.7 | 4.07 | 27 | 1 |
|  | 2w | 6.19 | 4.06 | 68 |  |
|  | 5w | 8.43 | 3.67 | 100 |  |
|  | 13w | 5.28 | 3.87 | 66 |  |
|  | 25w | 3.56 | 3.53 | 46 |  |
|  | | | | | |
| 31 | 1w | 2.62 | 4.69 | 74 | 1 |
|  | 2w | 6.36 | 4.07 | 147 |  |
|  | 5w | 2.87 | 4.45 | 112 |  |
|  | 13w | 2.82 | 4.65 | 114 |  |
|  | 25w | 3.71 | 4.81 | 120 |  |
|  | | | | | |
| 32 | 1w | 1.33 | 3.03 | 45 | 1 |
|  | 2w | 8.74 | 3.64 | 66 |  |
|  | 5w | 9.1 | 3.16 | 75 |  |
|  | 13w | 2.95 | 2.93 | 79 |  |
|  | 25w | 4.67 | 2.92 | 60 |  |
|  | | | | | |
| 33 | 1w | 3.29 | 5.41 | 63 | 1 |
|  | 2w | 7.77 | 5.17 | 66 |  |
|  | 5w | 5.6 | 5.26 | 67 |  |
|  | 13w | 5.28 | 5.24 | 59 |  |
|  | 25w | 5.59 | 5.23 | 62 |  |
|  | | | | | |
| 34 | 1w | 1.62 | 2.76 | 18 | 2 |
|  | 2w | 3.07 | 1.76 | 35 |  |
|  | 5w | 2.63 | 1.94 | 29 |  |
|  | 13w | 2.98 | 2.47 | 49 |  |
|  | 25w | 3.36 | 2.56 | 37 |  |
|  | | | | | |
| 35 | 1w | 2.94 | 4.2 | 39 | 2 |
|  | 2w | 2.91 | 4.3 | 49 |  |
|  | 5w | 3.64 | 4.13 | 47 |  |
|  | 13w | 4.67 | 4.34 | 52 |  |
|  | 25w | 5.67 | 3.79 | 34 |  |
|  | | | | | |
| 36 | 1w | 3.45 | 3.84 | 60 | 2 |
|  | 2w | 8.7 | 4.06 | 90 |  |
|  | 5w | 8.78 | 4.35 | 59 |  |
|  | 13w | 9.19 | 4.02 | 58 |  |
|  | 25w | 5.16 | 3.92 | 80 |  |
|  | | | | | |
| 37 | 1w | 2.05 | 3.96 | 65 | 2 |
|  | 2w | 9.32 | 3.79 | 168 |  |
|  | 5w | 8.95 | 4.05 | 173 |  |
|  | 13w | 8.03 | 3.86 | 158 |  |
|  | 25w | 8.13 | 4.65 | 164 |  |
|  | | | | | |
| 38 | 1w | 1.69 | 3.08 | 16 | 2 |
|  | 2w | 8.69 | 3.52 | 71 |  |
|  | 5w | 7.01 | 3.05 | 98 |  |
|  | 13w | 4.46 | 3.36 | 75 |  |
|  | 25w | 4.71 | 3.29 | 94 |  |
|  | | | | | |
| 39 | 1w | 2.79 | 3.1 | 74 | 2 |
|  | 2w | 3.27 | 3.15 | 63 |  |
|  | 5w | 3.14 | 3.2 | 74 |  |
|  | 13w | 3.23 | 2.9 | 71 |  |
|  | 25w | 3.96 | 3.12 | 63 |  |
|  | | | | | |
| 40 | 1w | 3.5 | 3.69 | 32 | 2 |
|  | 2w | 6.46 | 3.69 | 187 |  |
|  | 5w | 4.8 | 3.7 | 179 |  |
|  | 13w | 3.58 | 3.41 | 149 |  |
|  | 25w | 6.55 | 4.49 | 129 |  |
|  |  |  |  |  |  |
| 41 | 1w | 1.9 | 4.28 | 47 | 2 |
|  | 2w | 5.67 | 4.4 | 85 |  |
|  | 5w | 5.13 | 4.26 | 122 |  |
|  | 13w | 3.42 | 4.56 | 69 |  |
|  | 25w | 4.67 | 4.43 | 59 |  |
|  | | | | | |
| 42 | 1w | 5.37 | 5.09 | 44 | 2 |
|  | 2w | 7.6 | 4.98 | 147 |  |
|  | 5w | 7.65 | 5.28 | 80 |  |
|  | 13w | 5.93 | 4.13 | 110 |  |
|  | 25w | 7.94 | 5.39 | 144 |  |
|  | | | | | |
| 43 | 1w | 4.19 | 1.16 | 84 | 2 |
|  | 2w | 2.6 | 3.89 | 127 |  |
|  | 5w | 8.44 | 4 | 88 |  |
|  | 13w | 5.86 | 4.1 | 102 |  |
|  | 25w | 7.17 | 4.59 | 116 |  |
|  | | | | | |
| 44 | 1w | 3.3 | 3.13 | 77 | 2 |
|  | 2w | 6.39 | 2.89 | 97 |  |
|  | 5w | 4.12 | 3.7 | 126 |  |
|  | 13w | 11.2 | 3.42 | 152 |  |
|  | 25w | 5.78 | 3.64 | 123 |  |
|  | | | | | |
| 45 | 1w | 2.54 | 2.91 | 53 | 2 |
|  | 2w | 6.41 | 3.56 | 158 |  |
|  | 5w | 9.56 | 3.87 | 125 |  |
|  | 13w | 8.54 | 3.65 | 185 |  |
|  | 25w | 7.77 | 3.79 | 153 |  |
|  | | | | | |
| 46 | 1w | 3.41 | 4.34 | 37 | 2 |
|  | 2w | 5.18 | 3.53 | 46 |  |
|  | 5w | 2.47 | 4.07 | 46 |  |
|  | 13w | 3.17 | 4.36 | 63 |  |
|  | 25w | 4.5 | 4.54 | 60 |  |
|  | | | | | |
| 47 | 1w | 3.55 | 4.1 | 59 | 2 |
|  | 2w | 11.05 | 3.78 | 53 |  |
|  | 5w | 5.42 | 3.32 | 100 |  |
|  | 13w | 7.29 | 3.48 | 124 |  |
|  | 25w | 6.83 | 4.01 | 82 |  |
|  | | | | | |
| 48 | 1w | 4.41 | 3.49 | 58 | 2 |
|  | 2w | 9.04 | 3.71 | 197 |  |
|  | 5w | 5.17 | 2.16 | 188 |  |
|  | 13w | 8.04 | 2.72 | 129 |  |
|  | 25w | 7.63 | 2.51 | 130 |  |
|  | | | | | |
| 49 | 1w | 2.48 | 4.18 | 72 | 2 |
|  | 2w | 7.08 | 5.2 | 114 |  |
|  | 5w | 5.12 | 4.03 | 105 |  |
|  | 13w | 3.97 | 3.84 | 88 |  |
|  | 25w | 5.33 | 4.05 | 53 |  |
|  | | | | | |
| 50 | 1w | 1.05 | 4.08 | 36 | 2 |
|  | 2w | 5.08 | 4.03 | 77 |  |
|  | 5w | 2.51 | 3.64 | 76 |  |
|  | 13w | 9.1 | 3.89 | 79 |  |
|  | 25w | 3.85 | 3.2 | 66 |  |
|  | | | | | |
| 51 | 1w | 5.32 | 4.85 | 70 | 2 |
|  | 2w | 10.07 | 4.62 | 166 |  |
|  | 5w | 7.02 | 4.56 | 172 |  |
|  | 13w | 6.86 | 4.63 | 149 |  |
|  | 25w | 10.23 | 4.69 | 131 |  |
|  | | | | | |
| 52 | 1w | 2.09 | 2.24 | 22 | 2 |
|  | 2w | 6.26 | 2.51 | 78 |  |
|  | 5w | 1.97 | 2.38 | 56 |  |
|  | 13w | 5.72 | 2.89 | 66 |  |
|  | 25w | 7.88 | 3.04 | 86 |  |
|  | | | | | |
| 53 | 1w | 5.6 | 3.35 | 98 | 2 |
|  | 2w | 7.83 | 3.45 | 126 |  |
|  | 5w | 7.85 | 2.85 | 124 |  |
|  | 13w | 7.82 | 2.46 | 126 |  |
|  | 25w | 7.74 | 1.67 | 113 |  |
|  | | | | | |
| 54 | 1w | 2.15 | 3.58 | 34 | 2 |
|  | 2w | 12.09 | 3.56 | 80 |  |
|  | 5w | 3.02 | 3.4 | 78 |  |
|  | 13w | 2.96 | 3.55 | 74 |  |
|  | 25w | 6.49 | 4.38 | 82 |  |
|  | | | | | |
| 55 | 1w | 1.89 | 4.97 | 60 | 2 |
|  | 2w | 4.31 | 4.83 | 126 |  |
|  | 5w | 5.89 | 5.21 | 178 |  |
|  | 13w | 10.66 | 5.09 | 107 |  |
|  | 25w | 9.3 | 4.24 | 125 |  |
|  | | | | | |
| 56 | 1w | 1.89 | 3.91 | 31 | 2 |
|  | 2w | 2.13 | 4.29 | 59 |  |
|  | 5w | 3.43 | 4.06 | 47 |  |
|  | 13w | 3.01 | 4.23 | 75 |  |
|  | 25w | 3.53 | 3.54 | 46 |  |
|  | | | | | |
| 57 | 1w | 5.15 | 5.23 | 32 | 2 |
|  | 2w | 6.47 | 3.96 | 67 |  |
|  | 5w | 3.62 | 5.07 | 78 |  |
|  | 13w | 5.17 | 4.81 | 56 |  |
|  | 25w | 7.25 | 3.43 | 38 |  |
|  | | | | | |
| 58 | 1w | 2.55 | 3.42 | 36 | 2 |
|  | 2w | 4.6 | 3.25 | 56 |  |
|  | 5w | 2.64 | 3.36 | 54 |  |
|  | 13w | 1.76 | 3.49 | 52 |  |
|  | 25w | 3.37 | 3.55 | 58 |  |
|  | | | | | |
| 59 | 1w | 2.48 | 3.93 | 27 | 2 |
|  | 2w | 8.13 | 3.75 | 65 |  |
|  | 5w | 4.96 | 3.38 | 68 |  |
|  | 13w | 4.69 | 3.38 | 58 |  |
|  | 25w | 3.65 | 3.58 | 39 |  |
|  | | | | | |
| 60 | 1w | 3.4 | 4.33 | 57 | 2 |
|  | 2w | 11.59 | 4.27 | 114 |  |
|  | 5w | 3.6 | 3.51 | 128 |  |
|  | 13w | 3.42 | 3.78 | 108 |  |
|  | 25w | 4.98 | 3.56 | 95 |  |
|  | | | | | |
| 61 | 1w | 1.95 | 3.52 | 39 | 2 |
|  | 2w | 5.17 | 3.5 | 78 |  |
|  | 5w | 2.22 | 3.49 | 76 |  |
|  | 13w | 2.96 | 3.84 | 153 |  |
|  | 25w | 5.85 | 3.88 | 98 |  |
|  | | | | | |
| 62 | 1w | 5.15 | 3.04 | 83 | 2 |
|  | 2w | 10.28 | 3.02 | 135 |  |
|  | 5w | 3.32 | 3.05 | 150 |  |
|  | 13w | 3.68 | 3.43 | 153 |  |
|  | 25w | 4.16 | 4.24 | 116 |  |
|  | | | | | |
| 63 | 1w | 3.42 | 4.64 | 65 | 2 |
|  | 2w | 5.15 | 4.54 | 102 |  |
|  | 5w | 10.34 | 4.95 | 140 |  |
|  | 13w | 5 | 4.29 | 154 |  |
|  | 25w | 6.9 | 4.3 | 149 |  |
|  | | | | | |
| 64 | 1w | 3.1 | 4.51 | 51 | 2 |
|  | 2w | 8.67 | 4.61 | 65 |  |
|  | 5w | 4 | 4.4 | 76 |  |
|  | 13w | 4.97 | 4.23 | 78 |  |
|  | 25w | 6.57 | 3.77 | 91 |  |
