# Supplementary material for: Analysis of the efficacy of splenic artery superselective embolization in cirrhosis with hepatocellular carcinoma
Source: PLoS One. 2025 May 28;20(5):e0323829. doi: 10.1371/journal.pone.0323829 (PMC12119008; doi:10.1371/journal.pone.0323829)
Supplement: Attachment — Submitted filename: Response to reviewers.docx [file pone.0323829.s003.docx]

**Manuscript No.:** PONE-D-24-23322

**Title: Analysis of the efficacy of splenic artery superselective embolization in cirrhosis with hepatocellular carcinoma**

Dear editor，

Thank you very much for your reviewing and replying to our manuscript (PONE-D-24-23322). We would like to express our sincere gratitude to the editors for their comments. We replied point by point to the comments, revised the manuscript accordingly and the changes were highlighted in red in the revised manuscript. We sincerely hope this revised manuscript is more acceptable for publication.

Thank you again and look forward to hearing from you soon.

Yours sincerely,

Hong-Tao Hu

Response

1. Please ensure that your manuscript meets PLOS ONE's style requirements, including those for file naming. The PLOS ONE style templates can be found at 
https://journals.plos.org/plosone/s/file?id=wjVg/PLOSOne_formatting_sample_main_body.pdf

Thank you for your kind reminder, we have modified the manuscript.

2. Thank you for stating the following in your Competing Interests section:  
"The authors denies that they have any intention to obtain any financial interests."

Thank you for your kind reminder, we have added the information in Competing Interests section

3. We note that your Data Availability Statement is currently as follows: All relevant data are within the manuscript and its Supporting Information files.

Please confirm at this time whether or not your submission contains all raw data required to replicate the results of your study. Authors must share the “minimal data set” for their submission. PLOS defines the minimal data set to consist of the data required to replicate all study findings reported in the article, as well as related metadata and methods (https://journals.plos.org/plosone/s/data-availability#loc-minimal-data-set-definition).

For example, authors should submit the following data:

- The values behind the means, standard deviations and other measures reported;

- The values used to build graphs;

- The points extracted from images for analysis.

Thank you for your kind reminder, we have uploaded the data. The values behind the means, standard deviations and used to build graphs have shown in table 4. The raw data is presented in the supplementary material.

4. PLOS requires an ORCID iD for the corresponding author in Editorial Manager on papers submitted after December 6th, 2016. Please ensure that you have an ORCID iD and that it is validated in Editorial Manager. To do this, go to ‘Update my Information’ (in the upper left-hand corner of the main menu), and click on the Fetch/Validate link next to the ORCID field. This will take you to the ORCID site and allow you to create a new iD or authenticate a pre-existing iD in Editorial Manager. Please see the following video for instructions on linking an ORCID iD to your Editorial Manager account: <https://www.youtube.com/watch?v=_xcclfuvtxQ>

Thank you for your kind reminder, we have created an ORCID ID, Hong-Tao Hu：0000-0002-5432-4014

5. Please upload a copy of Figures 3, 4 and 5, to which you refer in your text on page 4 and 5. If the figure is no longer to be included as part of the submission please remove all reference to it within the text.

Sorry for confuse you, the Figures 3, 4 and 5 should be Fig.2C,D,E

6. Please review your reference list to ensure that it is complete and correct. If you have cited papers that have been retracted, please include the rationale for doing so in the manuscript text, or remove these references and replace them with relevant current references. Any changes to the reference list should be mentioned in the rebuttal letter that accompanies your revised manuscript. If you need to cite a retracted article, indicate the article’s retracted status in the References list and also include a citation and full reference for the retraction notice.

Thank you for your kind reminder, the reference is correct.
