# Supplementary material for: Analysis of the efficacy of splenic artery superselective embolization in cirrhosis with hepatocellular carcinoma
Source: PLoS One. 2025 May 28;20(5):e0323829. doi: 10.1371/journal.pone.0323829 (PMC12119008; doi:10.1371/journal.pone.0323829)
Supplement: Attachment — Submitted filename: Response to Reviewers2.docx [file pone.0323829.s004.docx]

Dear Reviewer #1,

Thank you very much for your valuable comments and suggestions. Your feedback is instrumental in enhancing the quality of our study, and we appreciate the opportunity to address each of your points in detail below:

1. Sample Size and Power Calculation

We acknowledge your concern regarding the small sample size, which was indeed due to the specificity of the cohort. We have now included a power calculation in the Methods section to demonstrate the statistical sufficiency of our sample size in supporting the conclusions drawn from our study.

2. Emphasizing the Need for PSE in the HCC Subgroup

We agree with your observation regarding the importance of evaluating PSE in the HCC subgroup. We have expanded the Introduction to better highlight the clinical significance of applying PSE to HCC patients. Additionally, we now elaborate on the unique challenges posed by portal hypertension and hypersplenism in this specific cohort, emphasizing the implications for clinical decision-making.

3. Effect of Liver Function on PSE

In response to your suggestion, we have added further details in the Discussion section on the potential impact of liver function on PSE efficacy. We also describe the steps we took in our study to minimize or standardize these effects, providing clarity on how this variable was managed.

4. Clinical Relevance of Postoperative Adverse Effects

Thank you for pointing out the importance of discussing postoperative adverse effects. In the Discussion, we have included a more detailed analysis of how these differences in postoperative outcomes could influence clinical practice, particularly in selecting the appropriate technique for individual patients.

5. Grammatical and Punctuation Errors

We have conducted a thorough review of the manuscript to address grammatical and punctuation issues, particularly in the Introduction, ensuring that our writing is clear and polished.

Dear Reviewer #2,

Thank you for your thoughtful comments and constructive feedback. Below are our responses to each of your suggestions:

Introduction:

We have added the necessary references to support key statements in the introduction, ensuring that all claims are appropriately backed by relevant literature.

Methods:

1. Data Source:

Thank you for pointing this out. We have clarified in the Methods section that the data used in this study were obtained from an institutional clinical database, not from a national administrative database.

2. Ethics Consideration:

We have rephrased and reformatted the ethics consideration section for better clarity and proper presentation.

3. Figure References:

We have corrected the figure reference order. Additionally, we have labeled Figure 1 and its sub-figures as Figure 1a, 1b, 1c, etc., for better clarity.

4. Interquartile Range and Median:

We have clarified the use of the interquartile range (IQR) and added the relevant mention of the median in the statistical methods section.

5. Pain Score Method:

We have specified that the pain score was assessed using the NRS (Numeric Rating Scale), a standardized method for pain assessment.

6. Liver Cirrhosis Diagnosis Reference:

We have added the appropriate reference regarding the diagnostic criteria for liver cirrhosis.

7. Spleen Infarction Volume Calculation:

We have added a reference for the calculation of infarcted spleen volume to support this method in our study.

Results:

1. Percentages in Results:

We have added percentages in the first paragraph of the Results section to provide clearer data representation.

2. Cohort Definition and Complication Rates:

We have better defined the cohort and included the overall complication rate in the first paragraph of the Results section for clarity.

3. Missing Numbers and Percentages:

We have added the missing numbers and percentages in the second paragraph of the Results section, as requested.

4. Pain Score Clarification:

We have revised the wording in the sentence regarding pain scores in Group A to ensure it is phrased clearly.

5. Post-treatment Mitigation Clarification:

We have deleted the relevant expression in view of its ambiguity.

6. Figure Quality:

We have reviewed the quality of the figures and attached them separately to ensure clarity and readability.

Tables:

1. Table 1 Caption:

We have updated the caption for Table 1 to: "Demographics and clinical characteristics of patients undergoing treatment," to provide a clearer description.

2. Age Variable:

We have corrected "agemean" to "Age" in Table 1 and included a note at the end stating that age is reported as mean and standard deviation, as recommended.

3. Hypertension Reporting:

We have simplified the hypertension reporting to only show the incidence of hypertension in one row, as suggested. The same approach has been applied to other conditions.

4. Etiology Category Clarification:

We have clarified the second category under "Etiology" in Table 1 to make it more understandable.

5. Laboratory Values:

We have included changes in laboratory values such as WBC and RBC for the overall cohort in the Results section.

6. Abbreviations in Table 1 Footnote:

We have expanded all abbreviations in the footnote of Table 1 for better clarity.

7. Table 2 Title:

We have improved the title of Table 2 to clearly state the data represented.

8. Splenic Abscess Reporting:

We have revised the results reporting for splenic abscess to ensure accuracy and clarity.

9. Table 4 Footnote:

We have expanded all abbreviations in the footnote of Table 4 as requested.

Discussion:

1. First Paragraph:

We have revised the first paragraph of the Discussion to provide a brief summary of the results rather than simply repeating them.

2. Citations in Paragraphs 2 and 3:

We have added the necessary citations to support statements in paragraphs 2 and 3 of the Discussion where literature findings or known facts are mentioned.

3. Discussion of Results in Paragraph 3:

We have revised the third paragraph of the Discussion to provide a more thoughtful analysis, comparing our findings with existing literature and discussing their implications.

4. Future Directions:

We have added a section on future directions, discussing the clinical significance of our findings and their potential impact on future research and practice.

Minor Comments:

1. Typographical Error:

We have corrected the typographical error in the last line of the Introduction on page 9 ("Emobilzationfor" to "Embolization for").

Thank you once again for your valuable feedback. We hope that these revisions address your concerns and improve the quality of our manuscript.

Sincerely,

Our Team
