# Supplementary material for: Analysis of the efficacy of splenic artery superselective embolization in cirrhosis with hepatocellular carcinoma
Source: PLoS One. 2025 May 28;20(5):e0323829. doi: 10.1371/journal.pone.0323829 (PMC12119008; doi:10.1371/journal.pone.0323829)
Supplement: Attachment — Submitted filename: Response to Reviewers-20250304.docx [file pone.0323829.s005.docx]

**Response to Reviewer #3**

Dear Reviewer #3,

We would like to express our sincere gratitude for your thorough evaluation of our manuscript, titled “Analysis of the efficacy of splenic artery superselective embolization in cirrhosis with hepatocellular carcinoma.” Your comments and suggestions have been most helpful, and we have revised the manuscript accordingly. Below is a point-by-point response, with references to where the revisions have been made.

### 1. ****Additional Details on Embolic Agent Dosage and the Use of Microcatheters****

**Reviewer’s Comment**

“In the specific procedure of splenic artery embolization, although the steps are described, additional details on the embolic agent dosage and the use of microcatheters could be added. For example, it would be useful to discuss why 710–1000 μm gelatin sponge particles were chosen, how the size of these particles affects the embolization outcome, and the advantages of using these particles compared to other embolic materials such as polyvinyl alcohol (PVA) particles or metallic coils.”

**Response**
We have expanded the “Materials and Methods” section to include more details about our embolization strategy:

**Rationale for 710–1000 μm Gelatin Sponge Particles**

1. These particles are suitably sized to occlude small-to-medium branches of the splenic artery, thereby achieving an approximate 30–50% splenic infarction while minimizing deep penetration into very small vessels.
2. Compared with smaller particles (<300–500 μm), 710–1000 μm particles enable a more predictable partial infarction range; whereas larger particles (>1100 μm) may yield insufficient ischemia or irregular infarction.
3. Gelatin sponge is a degradable material, reducing the risk of overly extensive and permanent infarction or abscess formation.

**Comparison with Other Embolic Agents**

1. **PVA Particles (Polyvinyl Alcohol).** These are non-degradable and can lead to more prolonged occlusion, which may be less desirable in cirrhotic patients who already carry risks of infection and complications if a large splenic area remains infarcted over an extended period.
2. **Metallic Coils.** Typically used for permanent vessel occlusion (e.g., treating aneurysms), coils are not well-suited for precisely controlling partial splenic infarction. They often occlude larger trunk vessels, thereby reducing the ability to tailor the infarct percentage in the spleen.

**Use of Microcatheters**

1. In the superselective (Group A) cohort, a microcatheter was navigated into specific splenic artery branches. This allowed more precise embolization of targeted segments (e.g., the lower pole) and minimized the risk of inadvertent embolization to pancreatic or other collateral branches.

These clarifications appear in the revised manuscript under “Splenic Artery Embolization.”

### 2. ****Pain and Complication Management****

**Reviewer’s Comment**

“Pain and Complication Management: In addition to pain and nausea/vomiting, pain management strategies could be added, such as whether analgesics were used postoperatively and the effectiveness of pain relief.”

**Response**
In the revised “Materials and Methods” and “Discussion” sections, we have provided further details on postoperative pain control:

- **Postoperative Pain Management**
  - **Mild–Moderate Pain (NRS <4).** Nonsteroidal anti-inflammatory drugs (NSAIDs) or acetaminophen.
  - **Moderate–Severe Pain (NRS ≥4).** Short-term opioid analgesics (e.g., oxycodone).
  - Most patients reported significant pain relief within 48–72 hours.

We believe this standardized approach effectively manages pain while minimizing adverse reactions from opioid usage.

### 3. ****Further Exploration of the Relationship Between Fever Duration and Splenic Infarction****

**Reviewer’s Comment**

“Although the discussion mentions that pain and fever are common postoperative complications of PSE, further exploration could be done on the relationship between the duration of fever and splenic infarction. Related literature could be cited to explain how the duration of fever relates to embolization dose, embolization site, and individual patient differences.”

**Response**
We have expanded the “Discussion” to analyze the correlation between fever duration and splenic infarction ratio:

- Literature indicates that when the splenic infarction exceeds 50–70% of the total splenic volume, patients tend to experience prolonged fever and higher incidences of complications. We have cited Zhu et al. [References 13–14 in the revised manuscript].
- Individual factors (e.g., Child-Pugh classification, baseline inflammatory state, and infection risk) also affect fever.
- In our cohort, the embolized fraction was generally 30–50%, and patients typically experienced transient fever (<3–4 days) without splenic abscess formation. We have added a new paragraph and references on these findings.

### 4. ****Minor Grammatical and Expression Issues****

**Reviewer’s Comment**

“There are a few minor grammatical and expression issues, such as some sentences being wordy. It is recommended to streamline the language for better readability. Specifically, language optimization in the methods and discussion sections would help ensure clarity and conciseness.”

**Response**
We have carefully edited the manuscript, especially the “Materials and Methods” and “Discussion” sections, to remove redundancies and enhance clarity:

- Reduced lengthier or repetitive sentences.
- Used more active voice to improve readability.
- Ensured consistent terminology and abbreviations.

These revisions strengthen the overall flow and coherence of the paper.

### ****Conclusion****

We sincerely appreciate your thorough review and valuable comments. By incorporating your suggestions—adding technical details, clarifying management strategies, and expanding our discussion on fever duration—we believe the manuscript is now significantly improved. We hope these revisions meet your expectations and would be grateful for any further feedback you might have.

**Thank you once again for your time and consideration.**

**Sincerely,**
The Authors
