# Supplementary material for: Analysis of the efficacy of splenic artery superselective embolization in cirrhosis with hepatocellular carcinoma
Source: PLoS One. 2025 May 28;20(5):e0323829. doi: 10.1371/journal.pone.0323829 (PMC12119008; doi:10.1371/journal.pone.0323829)
Supplement: Attachment — Submitted filename: Response to Reviewers-20250318.docx [file pone.0323829.s006.docx]

Response to Reviewers

Dear Editor and Reviewers,

Thank you for your constructive feedback and the opportunity to revise our manuscript. We have carefully addressed all the comments and suggestions, and the revised manuscript now incorporates these improvements. Below, we provide a point-by-point response to the reviewers’ concerns.

Editor’s Comments

1. Grammatical Errors and Language Clarity:

- Response: We thoroughly revised the manuscript to correct grammatical errors and improve clarity. Key adjustments include sentence restructuring, proper hyphenation (e.g., “non-superselective”), and consistency in terminology. The revised manuscript has been professionally proofread by an English editing service to ensure compliance with PLOS ONE’s language standards.

2. Abstract Revisions:

- Revised Text:

- “Patients with hypersplenism and hepatocellular carcinoma” (hyphenation and phrasing adjusted).

- “The safety and effectiveness of the two methods were evaluated, along with changes in peripheral blood cells… Postoperative adverse events (AEs) were also compared.”

- “PLT and WBC counts remained statistically elevated compared to pre-PSE levels (P<0.05), while RBC counts showed no significant difference (P>0.05).”

- “The incidence of fever and pain in Group B was significantly higher than in Group A (P<0.05). However, superselective embolization demonstrated fewer post-procedural complications.”

3. Introduction Clarifications:

- Terminology: “Pleural/ascites” was revised to “pleural effusion or ascites.”

- Hypothesis Rephrased: “We hypothesized that superselective splenic artery embolization could reduce necrotic tissue volume and peripheral stimulation, thereby lowering the risk of clinical complications.”

4. Methods Section Revisions:

- Exclusion Criteria: “Distant specialties” corrected to “distant metastases.”

- CT Diagnostic Criteria: Restructured as bullet points for clarity:

- Widened hepatic fissures, wavy hepatic margins, gallbladder displacement, heterogeneous liver density, portal vein thickening, collateral circulation, and splenomegaly.

- Splenomegaly defined as a maximum splenic diameter >12 cm on CT.

- Grouping: “Group A underwent superselective splenic artery branch embolization, while Group B received conventional PSE.”

5. Additional Revisions:

- Ensured consistent use of past tense in the Methods section.

- Replaced informal terms (e.g., “But” → “However”) in Results/Discussion.

- Verified subject-verb agreement and statistical reporting (e.g., “were” for plural outcomes).

Key Improvements in the Revised Manuscript

- Abstract: Enhanced flow and clarity with standardized terminology.

- Introduction: Clarified clinical context and hypothesis.

- Methods: Detailed exclusion criteria, imaging protocols, and procedural descriptions.

- Results/Discussion: Improved readability, statistical rigor, and interpretation of findings.

- Conclusion: Emphasized clinical relevance and technical advantages of superselective embolization.

We sincerely appreciate the opportunity to improve our work and believe the revised manuscript addresses all concerns raised. Should further revisions be required, we are committed to addressing them promptly.

Thank you for your time and consideration.

Best regards,

Dr. Zhao and Co-authors

Note: This response letter adheres to academic conventions, including formal tone, precise terminology, and structured formatting. All changes in the manuscript are highlighted in the tracked version for transparency.
